# Malaria Falciparum: Relapse After a Decade

**DOI:** 10.7759/cureus.26730

**Published:** 2022-07-11

**Authors:** Shriya Doreswamy, Hussein Al Sudani

**Affiliations:** 1 Internal Medicine, Einstein Medical Center Montgomery, East Norriton, USA

**Keywords:** falciparum malaria, reactivation, delayed, relapse, malaria

## Abstract

Malaria is an infection caused by the Plasmodium malaria (PM) parasite. There are still cases of malaria that are reported in the United States on an annual basis. All these cases were a result of travelers who did not receive or follow their prescribed chemoprophylaxis, recommendations for avoiding mosquito bites while traveling, or relapsed dormant plasmodium. The malaria parasite can be transmitted by the bite of an infected female mosquito, through contact with infected blood products, or from mother to child during pregnancy through the placenta. It can take anywhere from 12 to 20 days for symptoms to appear, but there are cases of delayed development and/or relapse that can occur up to 13 years after the infection. We report a 31-year-old female with a history of malarial infection in Liberia, which had been treated ten years prior to her arrival in the United States. She presented to the hospital with abdominal pain, fever, and headache. She was eventually diagnosed with plasmodium malaria infection relapse and treated with a 14-day course of primaquine 300 mg daily, with the symptoms resolving a few days after. We believe her malarial infection was caused by a dormant malarial parasite that evaded the immune system and relapsed without having a risk factor for relapse or re-infection 10 years after her original infection.

## Introduction

Plasmodium malaria (PM) is a parasite that has been recognized as the cause of malaria since around 2000 years ago. It has previously been observed that there is a relationship between the parasite's 72-hour lifecycle and the development of paroxysms of chills and fever when compared to the parasite's 48-hour lifecycle, which later led to the separation of findings between different parasites of malaria [1]. More than 1000 cases are reported in the United States each year, with all these cases occurring in travelers who did not receive or adhere to prescribed chemoprophylaxis, did not follow recommendations for mosquito bite prevention when traveling, or infections that persisted as they were not properly treated [2]. Transmission of the malaria parasite can be through the bite of an infective female mosquito, accidental blood product transfusion containing malaria parasite from a donor infected with malaria, or congenital transmission [3]. The duration between the time of transmission and the appearance of symptoms can take about 12 to 20 days (about three weeks). However, there are cases of delayed development and/or relapse that can occur up to 13 years after the infection [4,5]. PM infection can remain dormant in the liver in a form known as “hypnozoite” which is a stage in the life cycle of P. vivax and P. ovlae, this dormant form can be reactivated by a stimulus including the febrile illness associated with acute malaria or a different febrile infection and ultimately causing disease relapse, even after appropriate treatment of the blood-stage infection [6]. Conditions that were observed to have an association with PM relapse included loss of semi-immune state following splenectomy, during pregnancy, organ transplantation, and intravenous drug use through needle sharing [7-9]. In addition, malaria can be acquired from being around airports, which is known as “airport malaria” [10]. Although the exact mechanism of the parasite's persistence or recurrence for years before it causes any symptoms is not completely clear, there are reports that Plasmodium falciparum (P. falciparum) evades the immune response by a mechanism called “antigenic variation”, where the parasite can alter the surface proteins expressed that are usually targeted by the immune response. This can result in a phenotypically different population that can escape the host immune response and thereby prolonging the infection time and appearance of symptoms [11].

## Case presentation

A 31-year-old African American woman with a history of latent tuberculosis treated with three-month therapy and a malaria infection presented to our hospital in August 2021 with complaints of abdominal pain, headache, and fever. Her fever began three to four days prior to her presentation and was associated with two episodes of nausea and vomiting at home.

She lived at home with her husband and two children, ages five and seven years old, all in good health. She worked as a caretaker and claimed to have no other sick people in her life. She did not have any pets at home and did not interact with any other animals. She had not been camping, hiking, or participating in any other outdoor activities. She denied visiting other states or leaving the country since her arrival in 2014 from Liberia, where she has not returned since. She had malaria in Liberia about ten years ago and was treated with medications that she does not remember.

**Figure 1:**
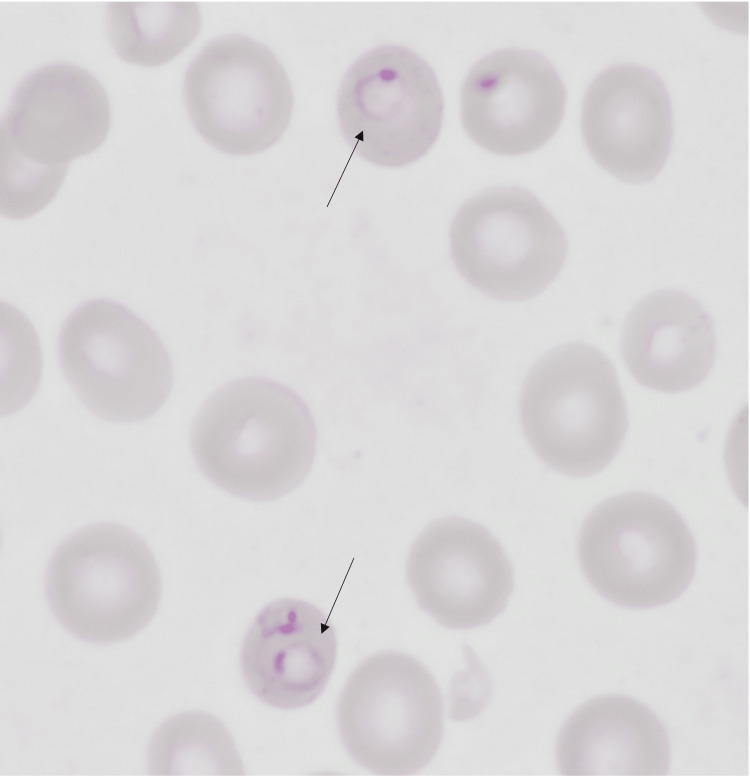
Black arrow shows intraerythrocytic ring form parasite of P. falciparum “trophozoite” in peripheral blood smear

On admission, the physical examination revealed a temperature of 37.8 degrees Celsius and tachycardia with a regular rhythm, but no other relevant findings. The white blood cell (WBC) count was 3.2× 10^9^/L (normal range 4.5-11.0 × 10^9^/L), hyperbilirubinemia with indirect bilirubin at 2 mg/dl (normal range 0.2-0.8 mg/dL), hemoglobin of 9.4 g/dL ( normal range 12.1-15.1 g/dL), MCV was 85 fl (normal range 80-100 fl), and platelet count was 43×10^9^/L (normal range 150-400 × 10^9^/L). A blood smear revealed intraerythrocytic parasitic elements as in (Figure 1). Polymerase chain reaction (PCR) testing confirmed the diagnosis of P. falciparum. She also had an abdominal ultrasound that revealed mild to moderate splenomegaly. She was seen by an infectious disease specialist, who recommended treatment with oral primaquine 300 mg for 14 days. She was later discharged from the hospital and followed up with her primary care provider within a week with a report of the resolution of her symptoms.

## Discussion

P. falciparum can survive in humans for long periods of time causing chronic infection. The longest reported is 13 years [5,12]. An immune response may be responsible for keeping the parasite's erythrocytic life cycle under control at low concentrations. Despite the lack of evidence for prolonged latency or an exo-erythrocytic parasite stage for P. falciparum, it is likely that the whole life cycle of these parasites occurs in the vascular spaces within the placenta or spleen. Additionally, P. falciparum has an array of complex immune evasion strategies, including the differential serial expression of var genes producing Plasmodium falciparum erythrocyte membrane protein 1 (PfEMP1), the primary surface antigen of the blood-stage, a process that is known as the antigenic variation method [11,13]. 

Immigrants and refugees who have left malaria-endemic areas were observed to have persistent falciparum infections and a sub-microscopic parasite prevalence. One of the studies showed that 195 African migrants living in Italy in 2007 had a parasite prevalence by the findings of 62 individuals with malaria who tested positive utilizing molecular detection methods, 13 of whom were gametocytic; 14 of the 62 cases were verified to have microscopic findings, 13 of which were P. falciparum [14]. These individuals may serve as a vector for the spread of malaria infection further.

For blood products, the presence of subclinical parasitemia in migrants from areas where parasites are prevalent is an issue. Blood transfusion-transmitted Plasmodium falciparum malaria from migrants who have been away from their country for years has been documented [8,15].

It is critical to distinguish between relapse and reinfection to establish the best treatment strategy. This is because antimalarial medicine resistance is determined by factors such as location, type, and prior treatments. Although this patient had previously been treated for malaria, the treatment and duration were unknown. She has not been in a situation that would put her at substantial risk of a new malarial infection, such as an organ transplant, blood transfusion, recent travel history, or living near an airport. Furthermore, she did not have any of the factors that typically lead to relapse, such as being pregnant, taking drugs, undergoing an organ transplant, or having a history of splenectomy, by altering her innate immunity [7-9]. Also, previous studies have demonstrated a relation between malaria and latent tuberculosis infection as in our patient history. It was observed in patients with this combination of infections to have a balance between the production of inflammatory and anti-inflammatory cytokines which can produce immunological protection against severe malaria similarly to our patient case [16].

## Conclusions

The importance of reporting this case is to have a high index of suspicion when treating patients with prior history of malaria infection. Being outside of the infectious zone for a long period of time does not rule out the possibility of malarial infection, and the index of suspicion should remain high. In our patient's case, her history of malaria led to the acquisition of a peripheral smear, and rapid PCR testing confirmed her diagnosis. Furthermore, it was reported that relapses can occur in a condition that alters the host immune system, however, our patient did not have any of those conditions and she was a healthy young female.
